# Impact of Nutritional Information on Consumers’ Willingness to Pay for Meat Products in Traditional Wet Markets of Taiwan

**DOI:** 10.3390/foods9081086

**Published:** 2020-08-09

**Authors:** Shang-Ho Yang, Ardiansyah Azhary Suhandoko, Dennis Chen

**Affiliations:** 1 Graduate Institute of Bio-Industry Management, National Chung Hsing University, No. 145 Xingda Rd., South Dist., Taichung City 40227, Taiwan; bruce.yang@nchu.edu.tw; 2 International Master Program of Agriculture, National Chung Hsing University, No. 145 Xingda Rd., South Dist., Taichung City 40227, Taiwan; ardiansyahazhary@gmail.com; 3 Massey College of Business, Belmont University, 1900 Belmont Boulevard, Nashville, TN 37212, USA

**Keywords:** traditional wet market, food product’s label, nutritional information, willingness to pay

## Abstract

The application of nutritional labels provides information regarding the health and nutritional value of products and allows consumers to engage in healthier dietary habits. However, not all types of retail markets provide full nutrition information for meat products. Since there is no nutritional information for fresh meat products in traditional wet markets, this study aimed to investigate consumer purchasing intention and willingness to pay (WTP) for this nutritional information in Taiwanese traditional wet markets. A total of 1420 valid respondents were examined using the random utility theory to explain consumer purchasing intention and WTP for nutritional information. Results showed that most (over 60%) consumers in traditional wet markets have positive purchasing intent for meat products with the nutrition information provided. Furthermore, the nutrition information in traditional wet markets significantly boosts consumers’ purchasing intention and WTP when consumers have a personal health awareness on meat, have proficient experience in buying meat, and continuously receive information from health-related media. Specifically, consumers’ shopping background and their level of health consciousness would be the key factors that would alter their WTP, if provided nutritional claims.

## 1. Introduction

A healthy lifestyle awareness regarding healthy eating habits among consumers is rising in these recent times. Nowadays, consumers are starting to think about the healthiness of food as one of the most important attributes and are starting to buy more products that positively relate to their health [1]. Additionally, unhealthy food selection leads to health issues such as diabetes, hypertension, and other non-communicable diseases (NCDs) [2,3,4]. Therefore, consumers are starting to be more selective when choosing their foods [5], and Taiwan had risen to be one of the top countries whose citizens think health is the most important factor when purchasing foods in the market [6].

Taiwanese consumers’ behavior in selecting food is consistent regardless of buying food at restaurants or for cooking at home. Moreover, it is widely believed that making food at home is associated with healthier food [7] and better life quality [8]. Thus, on many occasions, Taiwanese consumers are still keen on cooking at home. The habits of the Taiwanese are conducive to the prevalence of them visiting traditional markets because home cooks often visit there to purchase the freshest ingredients. However, cooking at home may be decreasing due to many factors, one of which is a lack of access or limited information regarding choosing healthy food products [9], especially in Taiwan [10]. Accordingly, this correlates with the decline of people visiting Taiwan’s traditional markets [11].

Taiwan’s traditional markets are quite popular among food buyers. These markets are often referred to as traditional wet markets [10,11] because they are often damp as a result of melting ice at meat stands and a sprinkling of water at vegetable vendors [12]. Nevertheless, buyers are still willing to frequent them despite their modest environments. Traditional wet markets are often visited by consumers of varied professions to buy foods, ranging from the elderly to managers of hotels and restaurants, particularly when seeking fresh products [13,14,15]. Traditional wet markets have other strengths that outshine other types of markets. These strengths include freshness [16], quality, social benefits (personal trust with buyers, buying–selling dialogue, and personal connectivity) [17,18,19,20], and the bargaining experience that saves money [12,21,22,23]. There are more than 50,000 merchants located in roughly 650 traditional wet markets across Taiwan [24], and their sales account for over $5.4 billion (3.84%) [25]. On the other hand, the vast emergence of hypermarkets (e.g., Carrefour and Costco) and the trend of younger Taiwanese consumers (which comprise approximately one-third of the population) who prefer to eat out put traditional wet markets’ continued popularity at stake [26,27,28,29]. Favorably, there are still distinctive products in traditional wet markets that can offset this worry and can possibly sustain their superiority relative to products in the more modern hypermarkets.

For example, meat products consistently attract consumers in Taiwanese traditional wet markets. People would rather buy meat in traditional wet markets because of its freshness, the flexibility in choosing particular meat parts, and lower costs [21]. Meat products in traditional wet markets draw more attention than other markets, resulting in the fact that up to 50% of the items sold in traditional wet markets are meat [16]. However, a significant risk remains that meats are still often connected with occurrences of non-communicable diseases [30,31]. Thus, consumers need tools to guide them to be healthy and to support their decision at the point of purchase.

Food labels might be able to help because food labels are believed to be a marketing tool and information strategy that eventually impacts consumers’ perceptions of food quality [32,33]. Additionally, if food labels regarding quality are applied to a product, they may create positive outcomes such as a willingness to pay significantly more or the ability to lure the consumers into becoming loyal buyers [34,35,36,37,38,39,40]. In addition, to address the healthier choice issue on consumers, nutritional information could help sellers target this better. Nutrition information is one of the helpful attributes of food labels that are used in many countries around the world, including Taiwan, to help consumers in deciding what to buy and to develop health-conscious food choices [41,42,43,44]. It has also been suggested by the Food and Drug Administration (FDA) and the United States Department of Agriculture (USDA) to apply this method. Food labels with nutrition information give easier information related to nutrition content and health in any food products corresponding to the food guide pyramid [45,46,47,48,49]. Furthermore, products with nutrition information should have a higher consumer willingness to pay (WTP) at the time of purchasing [50]. However, the benefits and utilization of nutrition information have not been realized among meat products in traditional wet markets.

Previous research studies involving traditional wet markets have been commonly focused on their management, marketing, and pricing strategies in comparison to other types of markets. Research regarding the attributes of consumers’ behaviors in traditional wet markets has not been widely observed [51,52]. The positive impact of nutritional information on consumers’ preferences about food products has been researched earlier in menus [53,54], supermarkets [55,56,57], grocery stores [58], cafés [59], and restaurants [60]. In addition, for specific products like meat, the effect of increasing purchasing power has been found to be similar in packaged meats in supermarkets [61,62], processed meats in hypermarkets [63,64], and meats at restaurants [60], which later increased buyers’ preferences. Therefore, this study fills this gap in research regarding nutritional information, as it has not yet been applied to traditional wet markets.

This study used an open-ended contingent valuation method to elicit consumers’ valuation on non-existing product attributes in the real market by asking them to what extent they are willing to pay extra in hypothetical markets [65]. Since nutritional labels are quite rare (or even not yet available) in the real traditional wet market environment, respondents evaluated the WTP of meat products with nutrition information like they do with non-market goods. Since this model follows an open-ended method, it is commonly used for this kind of research [66,67]. This research also adopts the random utility theory (RUT) to understand consumers’ behavior based on their circumstances and their habits [68]. Thus, the main objectives of this study were to investigate the impact of consumers’ preferences of nutritional information on meat products in traditional wet markets of Taiwan and to assess their WTP meat products bearing nutritional information. The first hypothesis suggested that consumers would give positive feedback differently based on their demographic background, even when applied in traditional wet markets. The second hypothesis suggested consumers would want to pay more for meat products that command premium prices. This paper also examined what kind of consumers prefer more or less of these types of meat products, as nutrition information’s effect would be varied based on their demographic profile. In addition, an increased consumer purchasing tendency may be a result when compared to prior research that has been done in different market contexts [58,60,64].

## 2. Materials and Methods

Traditional wet market consumers are likely to be unfamiliar with nutrition information for fresh meat products, so this study aimed to investigate consumers’ WTP if provided such nutrition information. This study adopted the contingent valuation method to explain consumer WTP for nutritional claims’ impact on meat products in a hypothetical condition within a traditional wet market. The consumers were surveyed with an open-ended questionnaire. To observe each consumers’ utility in buying meat products with nutrition information, the RUT was adopted in the research. Details of the methodology are explained further in the following section.

### 2.1. Participants and Survey Design

The study was conducted between July and August 2015 in cities across Taiwan. The respondents were interviewed at Taiwan traditional markets and train stations. The instructions given for the questionnaire were thinking about pork belly products that provided nutritional information. Pork belly meat products were chosen, because it is one of the most prominent animal parts bought in the Taiwan markets. Furthermore, pork belly’s price was at approximately 110–150 NT$/600 g in 2015 [69]. Thus, we acknowledge that based on the market study, the price for pork belly meat products across Taiwan (from south to north) were within this price range. We designed the survey based on the price that was currently available in the market. In the end, we took the 130 NT$ as the middle price.

A total valid 1420 respondents were collected. The sortation of 1420 people then provided options of who was reported to know the market price or who did not know/not sure the market price. For the respondents who knew the market price, they were sorted in one of the following categories: (i) 110 NT$, (ii) 130 NT$, or (iii) 150 NT$. People who chose (iv) do not know/not sure the market price were put in the situation of 130 NT$ price. Randomization was automatically generated by SurveyMonkey. The final sample sizes were as follows: (i) 110 NT$ group (*N* = 467), (ii) 130 NT$ group (*N* = 223), (iii) 150 NT$ group (*N* = 102), and (iv) the do not know the market price group (*N* = 628). An example of how participants are categorized in each of the three groups are shown in Table 1.

Before going further into the questions, the respondents were asked several screening questions, namely: (1) “Have you been to any traditional wet market in the past 12 months?” and (2) “Have you purchased any fresh meat products at a traditional wet market in the past 12 months?” If the respondents chose “No, I have not” or “No, I do not remember” and “No, I have not” or “No, I do not know,” respectively, then they were be considered for further analysis. To know the WTP of nutrition information, these kinds of questions were necessary to reduce sampling bias.

As the WTP for nutrition information was treated as a dependent variable in Equation (5), there were questions based on many independent variables categorized into the following three groups. The first independent variable group was socio-demographic, which consisted of (1) gender, (2) age, (3) family number, (4) family income, (5) education level, (6) housewife status, (7) location of survey (north, central, or south), and (8) respondents’ origin (urban or rural). The second independent variable group was shoppers’ customs, which consisted of (1) frequency of cooking at home, (2) main-shopper habits, (3) time spent for shopping, and (4) when visiting the market. The last independent variable group was nutrition-related information. This group consisted of (1) safety certificate, (2) meat grade information (whether it was fairly important, important, or very important to the respondents), (3) nutrition and calorie label, (4) fat and lean ratio information, and (5) health media concern. A detailed explanation for each variable’s measurement is shown in Table 1.

### 2.2. Theoretical Model Used

In traditional wet markets, specific attributes may contribute to consumers’ final purchase decisions, e.g., the cleanliness of the atmosphere, the bargaining situation, or other attributes that relate to their habits such as visiting time to a market. All of these attributes can affect their final decision. The RUT describes a consumers’ utility given the alternatives of attributes. The RUT is the model for each individual’s utility given the same situation of research with various ranges of each individual’s behavior and individual story. In other words, the RUT can be used to capture personal mobility choices. It has a basic hypothetical thought that every consumer is a decision-maker and they can maximize their utility relative to his/her choices [68]. By using the RUT, the shopper’s utility from one product can be comprised of the product’s function of attributes [70]. Moreover, regarding the budget issue of shopper’s perception, prior literature has suggested picking the set of attributes that might enlarge consumers’ utilities [71].

In this research, consumers’ personal choices were determined by the following three types of independent variables: socio-demographic, shoppers’ customs, and nutrition-related information. Therefore, the derived RUT mathematical model was written in vector notation as:

(1) $$U_{\mathrm{ij}}=\beta_{k}X_{ijk}+\varepsilon_{ij}$$

where U_ij_ represents the utility of *i*th shopper for pork *j* with nutrition information, *β* represents a homogenous vector of coefficients in which located among consumers, X_ijk_ represents the *k*th attribute of pork *j* for the *i*th shopper, and ε_ij_ represents the random residual that is unknown deviation for the user *i*’s utility. 

### 2.3. Data Analysis

The consumers’ decision on meat products bearing nutrition information does not solely rely on the meat’s health perception, as it is also associated with the independent variables that consist of socio-demographic, shoppers’ customs, and nutrition-related information. Thus, this study observed these factors predicting a probability regarding whether consumers would like to pay extra for meat products bearing nutrition information in traditional wet markets. Therefore, the data were analyzed using the logit model with the probability to give a positive WTP:

(2) $$p=\mathrm{pr}(y_{i}=1|X_{i})=F\left(X^{\prime }\beta \right)=\frac{e^{X^{\prime }\beta }}{1+e^{X^{\prime }\beta }}=\frac{\exp\left(X^{\prime }\beta \right)}{1+\exp\left(X^{\prime }\beta \right)}$$

where y_i_ = 1 stands for the probability to give a positive WTP and X_i_ stand for independent variables such as socio-demographic, shoppers’ customs, and nutrition-related information. Moreover, ∂𝑝/∂𝑥𝑗 = 𝐹’(*x’β*)*β_j_* shows the calculation of the marginal effect in this logit model.

Furthermore, this study attempted to estimate how much WTP for nutritional information of meat products in traditional markets. Interval regression was utilized because it has a mathematical simplicity and asymptotic characteristics, which constrained the predicted probabilities to a range of 0–1 and forecast the probability of willingness to pay [72] for nutritional labeling on pork belly products. Regarding the known interval boundaries of WTP, the interval regression model set-up can be demonstrated as below:

(3) $$y_{i}^{*}=x_{i}^{\prime }\beta +u_{i}$$

(4) $$\mathrm{Pr}\left[a_{j}<y^{*}\leq a_{j+1}\right]=\mathrm{Pr}\left[y^{*}\leq a_{j+1}\right]-\mathrm{Pr}\left[y^{*}\leq a_{j}\right]=F^{*}\left(a_{j+1}\right)-F^{*}\left(a_{j}\right)$$

where $y_{i}^{*}$ is observed to be in the (J+1) mutually exclusive intervals $\left(-\infty ,a_{1}\right]$, $\left(a_{1},a_{2}\right]$, …, $\left(a_{J},\infty \right)$. Given the answers individuals gave in the survey, $y^{*}$ was found to lie in corresponding intervals, i.e., $y^{*}\leq 0$, 1$<y^{*}\leq 3$, $4<y^{*}\leq 6$, …, and $16\leq y^{*}$. The empirical specification for the WTP for nutrition information on meat products in traditional wet markets is as follows:

(5) $$\mathrm{WTP}\;\mathrm{for}\;\mathrm{nutrition}\;\mathrm{information}=\;y^{*}=\beta_{0}+\beta_{1}X_{1}+\beta_{2}X_{2}+\ldots +\beta_{23}X_{23}+\varepsilon$$

where the WTP for nutrition information is explained by twenty-three independent variables (grouped into “socio-demographic” variables, “shoppers’ customs” variables, and “nutrition-related information” variables) that are represented by $X_{s}$. Then, $\beta_{s}$ represent the parameters to be estimated, and *ε* denotes the unobserved error term.

## 3. Results and Discussion

### 3.1. Sample Distribution

The distribution of the sample is presented in Table 1. According to Table 1, the willingness to pay for pork products with nutrition information in Taiwan traditional wet markets was found to be approximately 5.16 NT$/600 g. The socio-demographic data showed that more than half of the shoppers were 41-year-old or older females who had at least an associate degree (education period at least 15.22 years). Due to this fact, the monthly household income averaged 65,470 NT$ (or 785,640 NT$ as an annual household income). Moreover, about 13% of total respondents are purely identified as a housewife at their family, and the average number of family members are about four people in a family. Finally, the respondents were distributed geographically as follows: close to half were from Northern Taiwan, and one quarter were from Central Taiwan. These demographic patterns were similar to the previous studies in the Taiwan market that most of the samples are occupied by women (50%), with respondents above 30 years of age, high school graduates or below in education, approximately 775,673 NT$ annual household income, and housewives accounting for 25% of the sample [22,73,74,75,76,77]. Therefore, the sample means were very close to the population means in income and other measured categories. This revealed that our sample results may have been a good representative for the overall market conditions—though our sample means were representative of the population means, they had no effect on the WTP estimation. However, being representative of the population means may imply that our WTP estimations were close to the market condition as well.

Shoppers’ customs suggested that they are likely to cook their meals daily. The data also suggested that half of the respondents were the primary buyers of groceries for the family, while the rest were just casual buyers. Furthermore, the schedule and the duration of the shopping indicated that roughly half of the shoppers liked to shop in the morning between 5 and 11 a.m. and liked to take 30–60 min to buy food. It can be presumed that these respondents went for this period because the composition of them consisted of females and housewives who would have had time in the morning and the evening. However, roughly 10% of the buyers are the people who like to spend over 1 h wandering around the traditional wet market. Lastly, nutrition-related information suggested that 73% of the respondents thought that meat safety is relevant when supported by a safety certificate. Half of them also reported that the meat grade as provided by the butcher as a potential service is important (47%) or very important (17%). The reason for this was that almost half of the respondents were eager to get health-related information. Lastly, less than 40% of the people believed the nutrition and calorie label items, as well as fat and lean ratio information on meat products, would increase their willingness to pay.

### 3.2. The Probability of WTP an Extra

According to Table 2, the logit regression model fit well with these 23 independent variables, based on the indication of Wald χ^2^ test. Female shoppers showed a significant difference in wanting to pay extra if given additional nutrition information as compared to the males in this study. As shown in Table 1, the largest distribution of the sample was from the northern part of Taiwan. However, from Table 2, it can be seen that people from Central Taiwan had a higher WTP if provided nutrition information than the people from Southern or Northern Taiwan. This was supported by a previous researcher who mentioned that the penetration of the traditional market in the central area was stronger than other parts of Taiwan [78].

As seen in Table 2, the variables in shoppers’ customs did not indicate any significance in the results of estimated coefficients and marginal-effects likelihood. On the contrary, the variables within nutrition-related information were observed to show positive answers towards the importance of the potential service items provided by the butcher, namely meat grade information. The classification of meat grades—whether fairly important, just important, or very important—has a positive chance to add money to a consumer’s WTP. Nevertheless, the people who think meat grade information is very important, have the highest willingness to pay for nutrition label when compared to the other categories.

Moreover, from Table 2, it is seen that only nutrition and calorie labels would add to consumers’ WTP to purchase meat in the Taiwan traditional wet market, while fat and lean ratio information would not. These findings might link to health media concerns. Whereas, it is indicated that the respondents who frequently gain health information for themselves from mass media would give a more positive effect on WTP for nutrition information than the people who occasionally or never pay their attention to those platforms [79]. It can be said that females who think additional nutritional information can increase WTP are the groups of people who are aware of the healthy and food-borne disease. This finding was similar to the previous study that females tend to buy healthier meat than males in a market situation [80]. In short, the WTP for nutrition information is mostly affected by nutrition-related information that has an absolute impact.

### 3.3. The WTP for Nutrition Information on Pork

Based on Table 3, it can be seen that the respondents presented mostly as groups who knew the price. These groups represented 55% of the sample. However, 44% of consumers chose “do not know” or were not sure about the current meat price in Taiwan traditional markets. Since the groups who knew the price were based on the respondents’ knowledge about prices, this could be considered a random sortation for each group. Therefore, the largest group being those who knew the price value might have indicated that more people go to the lowest priced market or that more traditional markets are adopting the lower price market strategy. Within the groups of those who knew the price, the group with the lowest price value (110 NT$) dominated the sample, with 467 observations. They were followed by the other groups of people who selected 130 NT$ and 150 NT$, who had sample sizes of 223 and 102, respectively. From Table 3, it appears that the consumers who shopped for the lowest meat price might have had the highest WTP if given additional nutrition information.

#### 3.3.1. Ordinary Buyers

The people who chose the 110 NT**$** option had shorter education histories and could be assessed as ordinary buyers since they selected the lowest price (110 NT$) when they knew the price. They were regular people who would go to Taiwan traditional markets and might utilize additional information, such as nutrition and calorie labels and meat grade classification, as their decision-making factors. Though this was different than results from a prior study that stated that higher education people would pay attention to nutrition information more [81], this study showed that these Taiwanese people with less formal education would pay attention to nutrition information on meat products in traditional wet markets. When this group of people thought that this item was a very important potential service item provided by the butcher, they said that they would have a 4 NT$ more WTP for meat grade classification information. This group of ordinary buyers also said that they would increase their WTP by up to 2 NT$ if nutrition and calorie labels were added. Thus, this group’s dependability and WTP level on nutrition information were significant because they reported wanting the meat grade classification and nutrition and calorie label.

#### 3.3.2. Nutrition Information-Oriented Buyers

Ordinary buyers’ dependency on nutrition-related information was similar to the group who did not know the meat price. This group said that when they were provided with nutrition-related information attributes, they increased their WTP. This group could be considered nutrition information-oriented buyers since almost all the entire nutrition-related information factors showed significant and positive WTP. In addition, female consumers in this category reported that they would add about 1.3 NT$ to their WTP if provided nutrition labels. As described, females in this group did not know the price, so they relied on other information given by the butcher in nutrition information. This conclusion was supported by similar research found that that women are willing to pay an extra price for higher nutritional content in meat [80,81] since a woman’s decisions in the market often depend on external impulses [75]. Additionally, other findings have shown that this group raises its WTP by roughly 1 NT$ if the constituents are from an urban area and not a housewife. This finding might suggest these women are career women who live in big cities because they do not shop as often as housewives do. This is similar to prior research on career consumers that found that if products are given additional information, they might buy them more [82]. However, this result does not cover the fact that more career women exist than housewives nowadays. As stated before, the number of Taiwanese who are likely to eat out is growing [28,29], so these consumers tend to rely more on nutrition information when they do visit the market.

Moreover, nutrition information-oriented buyers who scored the meat grade classification as very important showed an approximately 0.60 NT$ higher WTP compared to ordinary buyers. This was consistent with previous studies that have shown that when nutrition information-oriented buyers care the most about meat grade, they are willing to pay more for the meat because they care greatly about quality, food safety, and health benefits [83,84,85]. This cluster also showed that they paid more attention to a safety certificate than the clusters who chose 110 NT$, 130 NT$ and 150 NT$. Thus, even though they did not know the price, if the meat product was given this kind of certificate, the WTP for nutrition information would rise by roughly 2 NT$. However, these consumers showed less WTP for nutrition and calorie labels than ordinary buyers, which means that they most likely perceived that safety and nutrition information were more important for their food choice. This is also supported by other prior studies that have stated nutrition information-oriented buyers’ preferences on nutrition information are based on their particular and personal nutrition utility satisfaction regarding meat products [62,86]. In general, nutrition information-oriented buyers are highly dependent on nutrition information, as they show a positive WTP in many of the nutrition-related information independent variables.

#### 3.3.3. Family-Oriented Buyers

The respondents who selected 130 NT$ as the meat price considered how many people lived in their house. The more family members they had, the more they were willing to pay at about 1 NT$. Additionally, this cluster was aware of the health media content through many mass media outlets since their WTP elicits approximately 2 NT$ more than the groups who do not know the price or chose 110 NT$. In addition, because they thought that meat grade was very important, they added an additional 4 NT$ to their WTP for this information. Thus, we can say that the shoppers who chose 130 NT$ were family-oriented buyers because they buy meat based on quality. Additionally, the management of food stock in their house was found to relate to their family member numbers. This finding aligned with similar prior research that found that a family with four or more members prefers to buy foods in traditional markets [77] and have a higher WTP if given additional nutrition information [87]. This is also supported by their knowledge about nutrition information from the mass media they access, so they know about the relevance of nutrition information for a healthy diet. Based on prior research, health media is one of the keys to increasing consumers’ preferences regarding nutrition information [79]. However, because they are family-oriented, these consumers may choose a standard price (130 NT$) because they need to manage their money for the welfare of the whole family. Therefore, this group was found to have a relatively higher dependability and WTP for nutrition information than ordinary buyers.

#### 3.3.4. Experienced and Proficient Buyers

The buyers who chose 150 NT$ were the people who relied on their previous buying experience in traditional wet markets. The effect of health content publication on mass media caused a rise of the WTP of the respondents in this group. It was shown that the health-related content, which they often obtained through mass media, increased their WTP by 6.5 NT$. Due to this habit, these consumers were seen as the only group who significantly relied on shoppers’ customs variables, such as their custom as the family’s shopper, their time management, and visiting time selection at the market. First of all, this group reported to prefer to be the primary shopper for their household, whether always or sometimes, and were willing to pay 10–12 NT$ more if they were given the nutrition information. Through these findings, it can certainly be observed that these people were more experienced than other categories and had more knowledge about the nutrition label, so they would prefer the highest price (150 NT$). For this reason, they were considered experienced and proficient buyers. This was aligned with a previous study that found that well-informed consumers show a high WTP on products bearing nutrition information [63]. Because of this reason, they would rather go to Taiwan traditional wet markets in the morning rather than the evening or night because they already know the best time to buy the best quality products [12,88]. They also seemed more efficient than other clusters because they did not prefer to stay in the market for more than 15 min since they already knew about the personal indicators that would affect their choices. It can be said that this category was more experienced about healthy diets because of their purchasing experiences including their knowledge from mass media [89]; thus, although they are a smart buyer, they rely on nutrition information and are willing to pay more for it.

### 3.4. The Estimation of Additional WTP for Nutrition Information

The results in this section describe the estimation of an additional WTP for nutrition information. These results were calculated by multiplying the average of each variable with the WTP in different consumers’ groups that showed significant results. Based on Table 4, the highest additional WTP of the socio-demographic group was found with consumers who had a higher family number in the household. These people were willing to pay up to roughly 134 NT$ when they chose 130 NT$. The next category of female shoppers, career shoppers, and urban shoppers who chose “do not know” showed a willingness to pay up to approximately 132 NT$. It can be said that people who chose 110 NT$ (who were previously considered ordinary buyers) suggested that the less education they have in their life, the more they need the nutrition level and the more additional money they add to the price. However, the overall WTP estimation was still negative for education. These results might indicate that consumers with a higher education would have a much less WTP than those who have a lower education. One reason that nutrition information-oriented buyers and family-oriented buyers were found to only have 2–4 NT$ additional budget might be that they think this 2–4 NT$ is a reasonable supplementary value for pork with nutritional information in traditional markets, if purchased at a middle price (130 NT$).

Following shoppers’ customs in Table 4, the top two additional WTPs were found not only from their behavior as a major buyer in the household but also their decision to choose a high-ranking price of 150 NT$. These experienced and proficient buyers who were the main shoppers were eager to buy pork with nutrition information until a price of 163 NT$. However, people in this group who were early bird shoppers and liked to spend less than one-hour shopping in traditional markets elicited a WTP of around 156 NT$. These experienced buyers (who were also primary shoppers) have the highest additional WTP. That is probably because they were either sometimes or always the decision-makers in their house for buying food, so nutrition information was important for them. Thus, when it came to the price, they did not hesitate to pay a premium, with an added price of up to 13 NT$. However, early-bird shoppers and speedy shoppers might have some personal preferences related to the time consumed and time visiting that interfere their decision in traditional markets. Thus, when facing pork with nutrition information, they were willing to pay a premium price, but only by about 7 NT$—about half of the added price of main shoppers’ attribute.

As for the nutrition-related information, the inferior additional WTP came from consumers who value safety certificate attributes, meat grade information (fairly important and important) attributes, and nutrition and calorie label attributes. These people were spread across consumers who chose 110 NT$ and 130 NT$ and did not know price options. Generally, they desired to pay roughly only an additional 2 NT$ for pork with nutrition information. This was consistent with a previous study that discussed meat product with a safety certificate and found that if consumers are provided with safety certificate information, they, in turn, have a higher positive WTP [90]. The middle place was the consumers who presumed that meat grade was very important for the supplementary vendor’s service. All buyers from all groups, except those who chose 150 NT$, said that they would pay an additional 4 NT$. Moreover, the elevated additional WTP was found on people from experienced and proficient buyers (chose 150 NT$), who are often concerned about health material learned from mass media. When they chose 150 NT$, they wanted to pay up to 157 NT$ for pork with nutrition information. The high additional WTP, up to 157 NT$, for nutrition-related information might be the result of their health knowledge gained from various platforms of mass or social media. The other groups with other nutrition-related information variables did not have a concern for health-related content, so their additional WTP only allowed them to upgrade by around 2–4 NT$. It can be stated that the more consumers have this access, the more they will increase their WTP, regardless of the attributes of nutrition-related information or price options, since they are considered health-conscious individuals [91]. 

**Figure 1:**
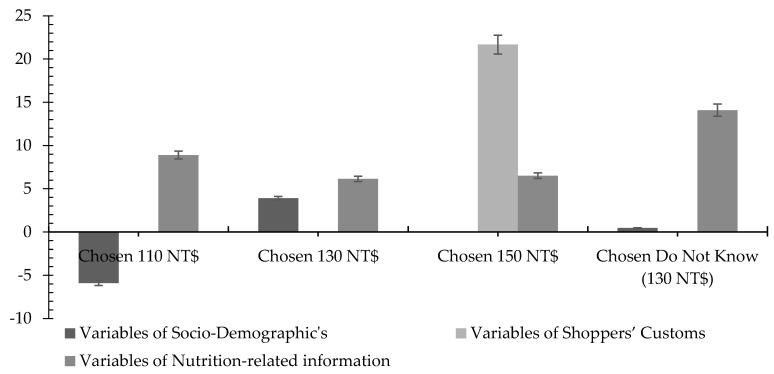
The estimation of WTP for nutrition information. Note: The error bars denote statistical significance at 5% significance. Source: Illustrated by this research.

**Figure 2:**
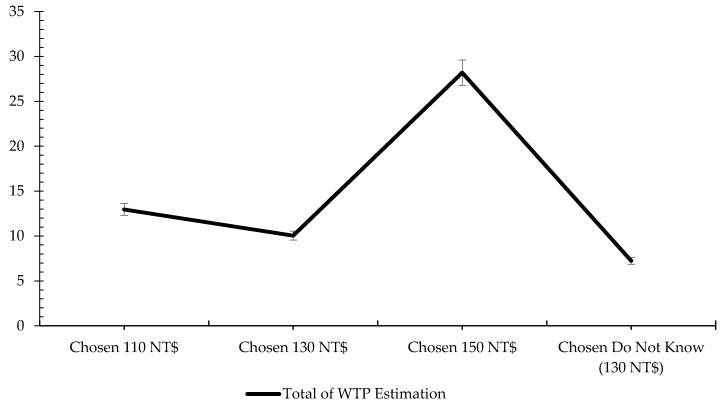
The total estimation of WTP for nutrition information. Note: The error bars denote statistical significance at 5% significance. Source: Illustrated by this research.

The total for each additional WTP for nutrition information is discussed further in the following visualization of Figure 1 and Figure 2. As seen in Figure 1, the additional WTP for each consumer group was combined based on three variable groups. Regarding the group who chose 110 NT$, it can be seen that nutrition-related information variables were important to them when deciding whether to buy pork with nutrition information. The people who chose 110 NT$ have significantly higher WTP for nutrition-related information (approximately 8 NT$) than the groups who chose 130 NT$ or 150 NT$. However, the total WTP of socio-demographic variables were negative among higher-education buyers for those who chose 110 NT$. This marked a significantly higher negative value than that of socio-demographic variables in the nutrition information-oriented buyers and family-oriented buyers’ groups. However, regarding shoppers’ customs variables, only the people who chose 150 NT$ showed a positive WTP, while the rest just revealed zero. From this result, it can be interpreted that ordinary buyers care more about nutrition-related information attributes when they visit traditional wet markets and buy pork with nutrition information. Those factors help them to better finalize their decision than the other two groups of independent variables. Nevertheless, as can be seen in Figure 1, family-oriented buyers’ nutrition-related information variables were not significantly higher than experienced and proficient buyers’, but these variables seemed to be the most important to them. Even though their socio-demographic variables were lower than the ordinary buyers’, they were higher than those of the nutrition information-oriented buyers and experienced and proficient buyers. This showed that when buying pork with nutrition information in a traditional market, both factors could impact a consumer’s additional WTP.

Moreover, in Figure 1, when combining the additional WTP of nutrition-information-oriented buyers’ (did not know the price) socio-demographic and shoppers’ customs variables, they did not have as much of an impact as much as nutrition-related information variables. It is because they both showed a value of almost zero. while nutrition-related information variables rose up to 15 NT$ and revealed significantly higher than ordinary buyers’ (8 NT$). It can be said that whenever people go to traditional markets not knowing the price and preferring nutrition attributes, they increase their WTP for pork with nutrition information. The reason for this is that they need more information to buy or simply because they prefer products with more nutrition-related information. Lastly, from Figure 1, we can also infer that experienced and proficient buyers’ shoppers’ customs variables showed significantly higher impacts than for the other price option groups, accounting for up to 22 NT$ when others accounted for nothing. This result could be seen as proof that when buyers have more experience and knowledge, their behavior on buying things in traditional markets impacts their preferences, especially on their additional WTP. Thus, providing pork with nutrition information is a good strategy to gain these people’s attention and money.

In addition, Figure 2 visualizes the total of all variables in every consumer group. According to Figure 2, the sum of the total variables showed significant differences across the four groups. This figure also shows a similar pattern in additional WTP, but one obvious result was that only three groups had the same 3 NT$ interval of the total. They were ordinary buyers, family-oriented buyers, and nutrition information-oriented buyers. Though they were significantly different from each other, their gap was still considerably smaller than each of their gaps with experienced and proficient buyers. When people chose 130 NT$ or did not know the price, their WTP rose until 140 NT$ and 137 NT$, which means that the people in these groups had an averagely similar thought pattern when facing pork with nutrition information. However, when they chose either the lowest or premium price, their WTP was added up to more than 10 NT$. Like ordinary buyers, their WTP went up to around 123 NT$. It can be said that when they chose lower price and were presented with pork with nutrition information, their WTP could reach middle or regular prices, which means that nutrition information gives them a chance to choose 130 NT$ in the future. However, for the experienced and proficient buyers, the addition of nutritional information can also capture a new possibility for pork belly’s price at around 175 NT$/600 g. We can conclude that these experienced and proficient buyers are likely to appreciate more nutrition information on meat products in traditional markets. Ultimately, a market vendor could enact a more optimal strategy by placing a premium price, since these experienced and proficient buyers are predisposed to pay an even higher price for nutrition information. This finding is similar to that of previous study that also concluded that additional improved-quality information for meat products made buyers willing to pay a premium price [63,71,91], even if it is over the current price.

## 4. Conclusions

Since many options, yet little health information, exist in traditional wet markets in Taiwan, consumers need guidance to decide which healthy products to choose. Nutrition information in traditional wet markets seems to be a tool in this modern era to help people pay more attention to healthy eating habits. Through this study, it was found that the nutrition information generally gives a positive impact throughout the independent variables and across several types of traditional wet market buyers. In conclusion, female shoppers, career woman shoppers, primary shoppers, morning shoppers, and big family shoppers reported their ability to increase their WTP for nutrition information on meat products in traditional wet markets. On the other hand, a less-educated buyer with longer shopping time in a market was found to not prefer nutrition information. Generally, variables in nutrition-related information were important in all of the groups. The variables in socio-demographic and shoppers’ customs might adjust to the consumers’ preferences. This adjustment is based on the influence of health-related-content (from mass media) and on their personal health preferences of meat. Thus, it can be said that when people think that the healthiness of food is one of the most important attributes, they are willing to pay attention to the additional labels.

Secondly, nutrition information showed a positive impact on the family-oriented buyers and experienced and proficient buyers since these two groups might watch and access the health-related content in mass media so they were varied from medium to high levels in terms of their reliability on nutrition information and WTP enhancement for the price selection of 130 NT$ and 150 NT$, respectively. The nutrition information-oriented buyers showed a more positive impact for nutrition-related information variables, because they were not equipped enough for the knowledge of nutrition labels and price (do not know the price). Thus, they reported requiring more instructions to finalize their decision. However, ordinary buyers showed a low reliability and WTP regarding nutrition information on meat products since they come from lower educational backgrounds and prefer to pay the lowest price (110 NT$).

Since there has not been much research observing traditional wet market consumers’ WTP concerning meat products bearing nutrition information, this research shows that the choice of applying nutrition information will bring positive feedback from the buyers and particular groups of consumers are even willing to pay more for this nutrition information. Thus, these findings will benefit traditional wet marketers in Taiwan if they apply these lessons to their marketing strategies. Lastly, these findings might be useful for the government to promote nutrition labels and other health-related content using mass media. Thus, the awareness of people to buy healthy food will be improved, including when they visit traditional wet markets, most of which currently do not provide additional nutrition labels. A limitation of this study was that the WTP estimation was done in hypothetical situations, and what a consumer intends to do (stated preferences in contingent valuation surveys) [92] and what they do in the real market could be different. Future research on this topic might approach different types of nutrition information such as nutrition claims, health claims, and traffic light labels, so meat consumers’ choices can be further described to help Taiwan traditional wet marketers build their marketing strategies.

## Figures and Tables

**Table 1:** The description of sample statistics of variables (*N =* 1420).

Variables		Mean	Description
**Dependent Variables**			
Positive WTP		0.61	BV = 1 if respondent is willing to pay any extra from 0 NT$ for nutrition information, 0 o/w
WTP for nutrition information		5.16	CV = Respondent’s WTP for nutrition information
**Independent Variables**			
**Socio-Demographic**			
Female		0.66	BV = 1 if respondent is female, 0 o/w
Age		41.07	CV = Years of age
Family number		4.14	CV = Number of members at home
Family income		65.47	CV = Monthly average household or family income
Education		15.22	CV = Years of education
Housewife		0.13	BV = 1 if occupancy of the respondent is housewife, 0 o/w
Northern Taiwan		0.49	BV = 1 if respondent is from Northern Taiwan, 0 o/w
Central Taiwan		0.28	BV = 1 if respondent is from Central Taiwan, 0 o/w
Urban		0.64	BV = 1 if respondent is from urban area, 0 o/w
**Shoppers’ Customs**			
Frequency cook at home		6.74	CV = Frequency to cook at home (the average number of times in one week)
Main-shopper (Always)		0.50	BV = 1 if respondent is always a major food shopper in the house, 0 o/w
Main-shopper (Sometimes)		0.32	BV = 1 if respondent is sometimes a major food shopper in the house, 0 o/w
Time spent (30–60 min)		0.50	BV = 1 if respondent spends 30–60 min to buy food in the traditional market, 0 o/w
Time spent (>1 h)		0.14	BV = 1 if respondent spends over 1 h to buy food in the traditional market, 0 o/w
Shopping time (5–11 a.m.)		0.43	BV = 1 if respondent purchases the food in the traditional market at 5–11 a.m., 0 o/w
Shopping time (11–5 p.m.)		0.22	BV = 1 if respondent purchases the food in the traditional market at 11 a.m.–5 p.m., 0 o/w
**Nutrition-Related Information**			
Safety certificate		0.73	BV = 1 if respondent examines safety certificate and meat safety are relevant, 0 o/w
Meat grade information	Fair	0.30	BV = 1 if respondent thinks the meat grade as a potential service provided by butcher is fairly important, 0 o/w
	Important	0.47	BV = 1 if respondent thinks the meat grade as a potential service provided by butcher is important, 0 o/w
	Very Important	0.15	BV = 1 if respondent thinks the meat grade as a potential service provided by butcher is very important, 0 o/w
Nutrition and calorie label		0.20	BV = 1 if respondent thinks the nutrition and calorie label item can increase the willingness to buy meat, 0 o/w
Fat and lean ratio information		0.36	BV = 1 if respondent thinks the fat and lean ratio item can increase the willingness to buy meat, 0 o/w
Health media concern		0.40	BV = 1 if respondent often watches the health-related content on TV or magazines, 0 o/w

Source: Grouped by this research. Note: (BV) and (CV) represent the binary and continuous variables, respectively, WTP represents the willingness to pay, and the o/w represents otherwise.

**Table 2:** The summary of logit model and marginal effect results for WTP an extra for nutrition information (*N* = 1420).

	Dependent	Positive WTP	
Independent		Coefficient	M.E.
**Socio-Demographic**			
Female		0.23 *	0.05 *
Age		0.00	0.00
Family number		0.04	0.01
Family income		−0.04	−0.01
Education		0.00	0.00
Housewife		−0.23	−0.05
Northern Taiwan		0.18	0.04
Central Taiwan		0.30 *	0.07 **
Urban		0.06	0.01
**Shoppers’ Customs**			
Frequency cook at home		0.00	0.00
Main-shopper (Always)		0.03	0.01
Main-shopper (Sometimes)		0.26	0.06
Time consumed (30–60 min)		−0.08	−0.02
Time consumed (>1 h)		−0.08	−0.02
Morning shopping time (5–11 a.m.)		0.08	0.02
Evening shopping time (11–5 p.m.)		0.04	0.01
**Nutrition-Related Information**			
Safety certificate		0.15	0.03
Meat grade information	Fairly Important	0.47 **	0.10 **
	Important	0.47 **	0.11 **
	Very important	0.63 **	0.14 ***
Nutrition and calorie label		0.59 ***	0.13 ***
Fat and lean ratio information		0.09	0.02
Health media concern		0.20 *	0.05 *
*Constant*		−0.40	
Log-Likelihood		−923.51	−923.51
Wald X^2^		51.28	
Pseudo R^2^		0.03	0.03

Source: Calculated by this research. Note: (***), (**), and (*) denote statistical significance at the 1%, 5%, and 10% significance, respectively.

**Table 3:** The estimation of WTP for nutrition information.

	Dependent	Chosen 110 NT$	Chosen 130 NT$	Chosen 150 NT$	Chosen Do Not Know (130 NT$)
Independent					
**Socio-Demographic**					
Female		0.25	−0.23	−1.88	1.37 **
Age		0.01	−0.02	−0.19	0.03
Family number		0.32	0.94 ***	−0.79	0.09
Family income		0.01	−0.03	0.00	0.01
Education		−0.39 **	−0.06	−0.39	−0.01
Housewife		−1.32	0.30	0.57	−1.86 **
Northern Taiwan		1.15	−0.18	−0.71	0.40
Central Taiwan		−0.01	−0.22	0.22	0.80
Urban		−0.28	1.46	−4.26	0.95 *
**Shoppers’ Customs**					
Frequency cook at home		−0.01	0.05	0.06	−0.04
Main-shopper (Always)		−1.06	−1.36	10.11 **	−0.05
Main-shopper (Sometimes)		−0.23	−1.19	12.61 ***	0.95
Time consumed (30–60 min)		0.31	1.04	−2.86	−0.78
Time consumed (>1 h)		0.37	1.12	−6.54 *	0.03
Morning shopping (5–11 a.m.)		−1.21	−0.60	5.49 **	0.67
Evening shopping (11–5 p.m.)		−0.43	0.95	3.73	0.36
**Nutrition-Related Information**					
Safety certificate		0.31	0.49	−0.80	2.00 ***
Meat grade information	Fairly Important	2.00	1.31	7.31	2.89 **
	Important	2.47 *	0.72	7.54	2.63 **
	Very important	4.05 ***	4.14**	11.45	4.63 ***
Nutrition and calorie label		2.38 **	1.29	3.43	1.94 ***
Fat and lean ratio information		0.83	−0.36	−3.97	0.01
Health media concern		0.43	2.00 **	6.51 ***	0.85
*Constant*		9.94 ***	*−2.89*	*−2.66*	−7.311 **
Observations (*n*)		467	223	102	628
Log-Likelihood		−1014.52	−380.42	−127.37	−1110.35
Wald X^2^		43.67	42.94	42.65	59.49
AIC		2079.04	810.84	304.73	2270.70

Source: Calculated by this research. Note: (***), (**), and (*) denote statistical significance at the 1%, 5%, and 10% significance, respectively.

**Table 4:** The estimation of additional WTP for nutrition information.

	Dependent	Average	Chosen 110 NT$	Chosen 130 NT$	Chosen 150 NT$	Chosen Do Not Know (130 NT$)
Independent						
**Socio-Demographic**						
Female		1	-	-	-	1.37
Family number		4.14	-	3.91	-	-
Education		15.22	−5.88	-	-	-
Housewife		1	-	-	-	−1.86
Urban		1	-	-	-	0.95
**Shoppers’ Customs**						
Main-shopper (Always)		1	-	-	10.11	-
Main-shopper (Sometimes)		1	-	-	12.61	-
Time consumed (>1 h)		1	-	-	−6.54	-
Morning shopping (5–11 a.m.)		1	-	-	5.49	-
**Nutrition-Related Information**						
Safety certificate		-	-	-	-	2.00
Meat grade information	Fairly Important	-	-	-	-	2.89
	Important	1	2.47	-	-	2.63
	Very important	1	4.05	4.14	-	4.63
Nutrition and calorie label		1	2.38	-	-	1.94
Health media concern		-	-	2.00	6.51	-
*Constant*		1	9.94	-	-	−7.23
Total of Additional WTP			12.96	10.04	28.19	7.23

Source: Calculated by this research.
